# Fine Particulate Air Pollution and Mortality in Nine California Counties: Results from CALFINE

**DOI:** 10.1289/ehp.8335

**Published:** 2005-09-01

**Authors:** Bart Ostro, Rachel Broadwin, Shelley Green, Wen-Ying Feng, Michael Lipsett

**Affiliations:** 1 California Office of Environmental Health Hazard Assessment, Oakland, California, USA; 2 University of California Davis, Davis, California, USA; 3 University of California San Francisco, San Francisco, California, USA

**Keywords:** air pollution, California, fine particles, mortality, particulate matter, PM_2.5_

## Abstract

Many epidemiologic studies provide evidence of an association between daily counts of mortality and ambient particulate matter < 10 μm in diameter (PM_10_). Relatively few studies, however, have investigated the relationship of mortality with fine particles [PM < 2.5 μm in diameter (PM_2.5_)], especially in a multicity setting. We examined associations between PM_2.5_ and daily mortality in nine heavily populated California counties using data from 1999 through 2002. We considered daily counts of all-cause mortality and several cause-specific subcategories (respiratory, cardiovascular, ischemic heart disease, and diabetes). We also examined these associations among several subpopulations, including the elderly (> 65 years of age), males, females, non-high school graduates, whites, and Hispanics. We used Poisson multiple regression models incorporating natural or penalized splines to control for covariates that could affect daily counts of mortality, including time, seasonality, temperature, humidity, and day of the week. We used meta-analyses using random-effects models to pool the observations in all nine counties. The analysis revealed associations of PM_2.5_ levels with several mortality categories. Specifically, a 10-μg/m^3^ change in 2-day average PM_2.5_ concentration corresponded to a 0.6% (95% confidence interval, 0.2–1.0%) increase in all-cause mortality, with similar or greater effect estimates for several other subpopulations and mortality subcategories, including respiratory disease, cardiovascular disease, diabetes, age > 65 years, females, deaths out of the hospital, and non-high school graduates. Results were generally insensitive to model specification and the type of spline model used. This analysis adds to the growing body of evidence linking PM_2.5_ with daily mortality.

Over the last decade, studies conducted over five continents have demonstrated associations between daily exposure to particulate matter (PM) < 10 μm in aerodynamic diameter (PM_10_) and premature mortality [U.S. Environmental Protection Agency (EPA) 2004]. The U.S. EPA promulgated ambient air quality standards for fine particles [those < 2.5 μm in diameter (PM_2.5_)] in 1997 and is currently considering revisions to these standards; however, relatively few studies have examined relationships of this pollutant class with mortality (Burnett et al. 2003; Schwartz 2003; U.S. EPA 2004). In addition, most studies to date have been conducted in the eastern United States, Canada, and Western Europe. Relatively few studies have been conducted in California, where particle sources, chemistry, size distribution, and temporal patterns of exposure are quite different. Specifically, existing evidence suggests that, in California, *a*) nitrates comprise a larger fraction of PM_2.5_ than they do in other regions, and *b*) mobile sources represent the predominant source of PM_2.5_, whereas a mix of mobile and stationary sources predominate elsewhere (Blanchard 2003). Moreover, in the Los Angeles air basin, peak PM_2.5_ exposures occur in both winter and nonwinter months.

In 1999, the U.S. EPA and the California Air Resources Board (CARB) embarked on a program to collect daily data on PM_2.5_ in many cities throughout California. We have obtained and linked daily readings of PM_2.5_ with mortality in nine heavily populated counties in California. The ability to explore hypotheses of association with adverse health in multiple cities has several distinct advantages. It enhances the power of the statistical analysis and reduces the likelihood of spurious results or publication bias that might result from the analysis of a single city (Anderson et al. 2005). In this article, we report the results of our analysis of the relationship between mortality and fine particles in California (CALFINE).

## Materials and Methods

### Mortality data.

Data on daily mortality were obtained for all California residents from the California Department of Health Services (CDHS), Health Data and Statistics Branch, for the period 1 January 1999 through 31 December 2002 (CDHS 1999–2002). Our study was limited to deaths occurring in nine California counties (cities where the monitors were located are in parentheses): Contra Costa (Concord), Fresno (Fresno), Kern (Bakersfield), Los Angeles (Los Angeles, North Long Beach, Azusa), Orange (Anaheim), Riverside (Riverside), Sacramento (Sacramento), San Diego (San Diego, Escondido, El Cajon), and Santa Clara (two in San Jose). Data were limited to deaths occurring in the decedents’ county of residence. Daily counts of total deaths (minus accidents and homicides) were aggregated. Using the *International Classification of Diseases, 10th Revision* (ICD-10) (World Health Organization 1993), total daily counts of deaths from respiratory disease (ICD-10 codes J00–J98), cardiovascular disease (ICD-10 codes I00–I99), ischemic heart disease (ICD-10 codes I20–I25), and diabetes (ICD-10 codes E10–E14) were also calculated.

We also calculated daily, all-cause mortality counts for the following subpopulations and mortality categories: *a*) age > 65 years, *b*) males, *c*) females, *d*) white non-Hispanic, *e*) black non-Hispanic, *f*) Hispanic, *g*) in hospital, *h*) out of hospital, *i*) less than high school education, and *j*) high school graduate.

### Pollutant and meteorologic data.

We obtained pollution data for the 4-year period 1999 through 2002 from multiple sources. Daily average PM_2.5_ data were obtained from the U.S. EPA’s Aerometric Information Retrieval System (AIRS) database. PM_2.5_ monitors were filter-based, ambient air samplers (model RAAS2.5-300; Andersen Instruments, Inc., Smyrna, GA).

This sequential sampler is designated as a federal reference method sampler for collection of PM_2.5_. There was only one monitor collecting daily PM_2.5_ data in each of the nine counties, except for Los Angeles, San Diego, and Santa Clara counties, which had three, three, and two monitors, respectively. Data from the nine counties represent nearly all locations of monitors in California that measured PM_2.5_ on a daily basis for large parts of 1999–2002. A substantial number of days were missing data, which varied by county and appeared to be fairly random, with a few exceptions. Specifically, in 1999 several of the counties had no data from January through March, and from March through December, Los Angeles and Riverside counties had data only every third day.

Data on gaseous pollutants, including carbon monoxide, nitrogen dioxide, and ozone, were obtained from the CARB air quality database for all nine counties. Most of the monitors for gases were part of the State and Local Air Monitoring Stations (SLAMS) network. All gases were reported as 24-hr averages, except ozone, which was reported as both an 8-hr average (1000–1800 hr) and as a 1-hr maximum.

For counties with multiple monitors, the daily average was calculated using all available data. To account for missing data among some of the monitors, we used a process similar to that described by Wong et al. (2001). The average was developed by *a*) calculating the mean for each monitor, *b*) subtracting the mean concentration of each monitor from the nonmissing daily values, *c*) calculating the mean of the available adjusted data, and *d*) adding back the grand mean of the data.

To allow adjustment for the effect of weather on mortality, we collected daily average temperature and humidity data at weather stations in each of the nine counties. Hourly temperature data were obtained from AIRS for all sites except Contra Costa and Santa Clara counties, for which data were obtained from the Bay Area Air Quality Management District and from Golden Gate Weather Services, respectively. All daily mortality, pollutant, and meteorologic data were converted into a SAS database (SAS Institute Inc., Cary, NC) and merged by date. This resulted in 4 years (1,461 days) of daily time-series data.

### Methods.

Counts of daily mortality are nonnegative discrete integers representing rare events; such data typically follow a Poisson distribution. Therefore, the analysis relied on Poisson regression, conditional on the explanatory variables. In the basic analytic approach, we used similar model specifications for each city, including smoothing spline functions for time trend and weather. We examined both penalized and natural spline models. The penalized spline model is a flexible, nonparametric approach using cubic splines and a term that penalizes the curvature of the smoothing function (Wood 2000). The “roughness penalty” controls the trade-off between a precise fit of the data and a smoothed function. The model then minimizes the sum of the squared deviations plus the penalty function to determine the amount of smoothing in the fit. The natural spline model is a parametric approach that fits piecewise polynomial functions joined at knots, which are typically placed evenly throughout the distribution of the variable of concern, such as time. The function is constrained to be continuous at each knot (Ruppert et al. 2003). The model also places two additional knots at the ends of the data, with the function constrained to be linear beyond these points. The number of knots used determines the overall smoothness of the fit. Previous analysis has indicated that different spline models generate relatively similar results (Health Effects Institute 2003). However, depending on the underlying data and model specifications, different splines might produce varying degrees of bias and efficiency in the regression estimates.

For the initial analysis of all-cause, cardiovascular, respiratory, and above-age-65 mortality, a penalized spline regression was used with R (R Development Core Team 2004). We incorporated a smoothed spline function of time, which can accommodate nonlinear and nonmonotonic patterns between time and mortality, offering a flexible modeling tool (Hastie and Tibshirani 1990). In addition, the smooth of time diminishes short-term fluctuations in the data, thereby helping to reduce the degree of serial correlation. Based on previous findings reported in the literature (e.g., Samet et al. 2000), the basic model included a smoothing spline for time with 7 degrees of freedom (df) per year of data. This number of degrees of freedom controls well for seasonal patterns in mortality and reduces and often eliminates autocorrelation. Visual inspection of the data indicated a spike in mortality in several of the cities in southern and central California during a 3-week period starting 17 December 1999. During this period, the actual number of cases exceeded the smoothed estimate. Therefore, for all of the regression models, we added a second smooth of time with 3 knots for this 3-week period.

Other covariates, such as day of the week and smoothing splines of 1-day lags of average temperature and humidity (each with 3 df), were also included in the model because they may be associated with daily mortality and are likely to vary over time in concert with air pollution levels. Previous studies have reported stronger associations of mortality with PM lagged 1 or 2 days or with cumulative exposures over several days. Therefore, in our primary analysis of PM_2.5_, we examined two different *a priori* lag structures: a 2-day average of lags 0 and 1 (lag 01) and a single-day lag of 2 days (lag 2). The county-specific results were then combined in a meta-analysis using a random effects model in Stata (StataCorp 2003). The meta-analysis focused primarily on all-cause mortality and on cardiovascular, respiratory, and elderly (> 65 years of age) mortality, because these categories have been the focus of previous time-series studies (Health Effects Institute 2003).

We also conducted several sensitivity analyses. First, we examined these same four outcomes using a similar specification, but with a natural spline model. For each county, we used lag 01 for PM_2.5_ and 4, 8, and 12 df/year for the smooth of time. Second, using lag 01 and penalized spline models with 7 df for the smooth of time, we examined other mortality groupings and classifications, including those for males, females, whites, blacks, Hispanics, high school and non-high school graduates, deaths occurring in and out of hospitals, ischemic heart disease, and diabetes. Finally, we examined the impact on the estimated coefficient of PM_2.5_ when gaseous pollutants were added to the penalized spline model (i.e., in two-pollutant models specified with PM_2.5_ and each of the gaseous pollutants).

All final results were calculated using R (version 1.9), and the results are presented as the percent change in daily mortality per 10 μg/m^3^ PM_2.5_. The percent change per 10 μg/m^3^ is simply the β-coefficient (times 1,000) from the Poisson regression.

## Results

Tables 1 and 2 provide the descriptive statistics for population, air quality, mortality, and meteorologic data from the nine counties. The populations in 2000 ranged from 661,645 in Kern County to 9,519,338 in Los Angeles County; the total in these nine counties accounted for 65% of California’s population in 2000. Mean daily mortality varied from 146 in Los Angeles County to 11 in Kern County. Mean daily PM_2.5_ levels ranged from 14 μg/m^3^ in Sacramento and Contra Costa Counties to 29 μg/m^3^ in Riverside County, exceeding the U.S. EPA annual average PM_2.5_ standard of 15 μg/m^3^ in six of the nine counties. Temporally, among the cities, PM_2.5_ was highly correlated with both nitrogen dioxide (mean *r* = 0.56; range, 0.38–0.66) and carbon monoxide (mean *r* = 0.60; range, 0.37–0.83), but only moderately and often inversely correlated with both 1-hr ozone levels (mean *r* = −0.14; range, −0.39 to 0.17) and 8-hr ozone levels (mean, −0.22; range, −0.47 to 0.12).

Table 3 summarizes the basic results for the meta-analyses for four mortality categories using penalized splines with two different lag structures. The results suggest associations between PM_2.5_ and all-cause, cardiovascular, respiratory, and elderly mortality. Point estimates of risk were particularly elevated for respiratory-specific mortality. Also, cumulative exposures of 2 days generated larger pooled effect estimates than did the single-day lags that were examined. Diagnostics indicated that autocorrelation was present over the entire data series for many of the counties when a simple smooth of time was used. The autocorrelation was eliminated, however, when the second smooth of time was included for the 3-week period starting 17 December 1999.

Table 4 summarizes the results for the meta-analyses for four mortality categories when similar models were used with lag 01 for PM_2.5_ and natural splines for the smoothers of temperature and humidity and three alternative smoothers of time. The results generally support, but are slightly lower than, those observed using penalized splines (Table 3), indicating associations with all-cause, respiratory, and elderly mortality and more modest associations with cardiovascular mortality. In addition, greater degrees of freedom for time trend tended to lower the effect estimates.

Table 5 summarizes the meta-analytic results for PM_2.5_ for different mortality categories and subpopulations using a penalized spline model and lag 01. The results suggest somewhat stronger associations of daily PM_2.5_ concentrations with mortality for diabetics, females, and whites. The association for deaths occurring outside of hospitals was demonstrated with greater precision than for those occurring inside hospitals. In addition, the point estimate for mortality among those who had not graduated from high school was more than twice that of those who had, with an association that was of marginal statistical significance (*p* < 0.10). Finally, in multipollutant models (using lag 01), the estimated PM_2.5_ coefficient was attenuated when the highly correlated pollutants—nitrogen dioxide and carbon monoxide—were added to the model but was not affected by the inclusion of either 1-hr or 8-hr ozone. However, for mortality among those > 65 years of age, the inclusion of any of the gaseous pollutants to the model did not affect the PM_2.5_ coefficient (data not shown).

## Discussion

In this time-series analysis in nine California counties, short-term exposures to PM_2.5_ were associated with increased daily mortality. These results appear to be relatively insensitive to the use of natural versus penalized spline model and the degrees of freedom in the smoothing functions for time, although both of these factors alter the effect estimates. Specifically, PM_2.5_ was associated with all-cause, cardiovascular, and respiratory mortality, as well as with deaths in persons > 65 years of age. PM_2.5_–mortality associations were particularly elevated among females, whites, persons who did not graduate from high school, diabetics, and those who died out of hospital.

Several earlier studies that examined associations between daily mortality and either PM_10_ or PM_2.5_ were reanalyzed for the Health Effects Institute (Health Effects Institute 2003). The reanalyses were conducted after the generalized additive models had been found to produce biased effect estimates and standard errors when default convergence criteria were used in S-Plus (Dominici et al. 2003). Regarding PM_2.5_, Schwartz et al. (1996) found statistically significant increases in mortality in their reanalysis of the Six Cities study using both natural spline [1.29% per 10 μg/m^3^ PM_2.5_; 95% confidence interval (CI), 0.88–1.70] and penalized spline (1.13%; 95% CI, 0.70–1.56) models with 4 df/year for time. Burnett et al. (2003) reexamined nonaccidental mortality from 1986 to 1996 in eight Canadian cities, using natural spline models with 2 df/year for time, and reported a 1.10% increase in mortality (95% CI, 0.35–1.85) per 10 μg/m^3^ of PM_2.5_. A reanalysis of another Canadian study found a nonsignificant increase in mortality (0.46% per 10 μg/m^3^ PM_2.5_) in Montreal from 1984 to 1993 (Goldberg and Burnett 2003). In a reanalysis of a time-series study in Santa Clara, California, Fairley (2003) reported a 2.75% increase (95% CI, 0.61–4.89) in nonaccidental mortality per 10 μg/m^3^ PM_2.5_ using a natural spline model with 9 df/year. The reanalyses of data from Detroit (Ito 2003) and Los Angeles (Moolgavkar 2003) using natural spline models demonstrated positive but nonsignificant increases in mortality of 0.79 and 0.55%, respectively, per 10 μg/m^3^ PM_2.5_. Finally, in a study in Mexico City, Mexico, PM_2.5_ was associated with a 1.4% (95% CI, 0.2–2.5) increase in daily mortality per 10 μg/m^3^ (Borja-Aburto et al. 1998).

Our effect estimate of about 0.6% per 10 μg/m^3^ PM_2.5_ for all-cause mortality is in the lower end of the range of these previous estimates. There are several possible explanations for the lower effect estimates. First, large exposure measurement errors were likely, owing to the use of one to three monitors to represent exposure in these counties, some of which extend over thousands of square miles. Therefore, assuming such measurement errors were nondifferential with respect to the populations at risk, the effect estimates would likely be biased downward. Second, the composition of PM_2.5_ in California, which in several of these counties is dominated by nitrates, may be less toxic, particularly to the cardiovascular system (Schlesinger and Cassee 2003). However, this hypothesis contrasts with the findings of one of the few studies to explicitly examine the effects of nitrates, which were associated with significant increases of mortality in Santa Clara County (Fairley 2003). Third, California residents may be less susceptible to the cardiovascular effects of air pollution, possibly due to differences in exercise and dietary patterns, or to active and passive smoking rates that are lower than national averages. Fourth, there may be geographic confounding related to some unknown and therefore unmeasured spatially varying factors. Finally, this could be a chance finding. The likely potential importance of measurement error, geographic confounding, and chance is suggested by the large variability in effect estimates among the nine counties. Such heterogeneity has also been reported in the analysis of the 90 largest U.S. cities (Samet et al. 2000). There is no obvious explanation for the different PM_2.5_–mortality associations in each county. This merits further study.

Of additional interest is the strength of the association of PM_2.5_ with respiratory mortality relative to that for cardiovascular mortality. Many previous studies [reviewed by Ostro et al. (1999)] report stronger effects for cardiovascular mortality, which may be due to *a*) the greater prevalence of circulatory disease (and therefore increased statistical power) and *b*) the likely attribution of cause of death as cardiovascular when there is uncertainty or when there is an underlying respiratory condition. It is often more difficult to detect associations between air pollution and respiratory deaths because the latter generally represent a small fraction of total mortality and are more likely to be ascribed to cardiovascular causes than vice versa. However, it is clear that PM_2.5_ and other PM metrics are associated with daily mortality from respiratory causes. For example, Penttinen et al. (2004), Zanobetti et al. (2003), Braga et al. (2001), and Ostro et al. (1999) all report stronger associations of PM with respiratory than with cardiovascular mortality. De Leon et al. (2003) reported that those with an underlying respiratory condition were more susceptible to the impacts of air pollution on nonrespiratory (e.g., circulatory or cancer-related) mortality. Associations have also been reported between PM_2.5_ and respiratory morbidity, including hospitalizations and emergency department visits for respiratory disease (Delfino et al. 1997; Ito 2003; Peel et al. 2005).

Our analysis also suggests that diabetics and those with less than a high school education may be at increased risk from exposure to PM_2.5_. Several previous time-series studies have reported that diabetics may be at increased risk from exposure to PM (Goldberg et al. 2001; Zanobetti and Schwartz 2002). Pope et al. (2002) reported that educational attainment was an important effect modifier in the association between long-term exposure to PM_2.5_ and survival. However, susceptibility to PM pollution is not likely to be affected by education per se, but rather by factors that might be associated with education, such as nutritional status, access to health care, occupation, psychosocial stress, and residential proximity to heavy traffic. On the other hand, most time-series studies to date have not reported a significant effect modification by socioeconomic status (Samet et al. 2000; Schwartz 2000). We also found, as have others, a better model fit for PM_2.5_ for deaths occurring out of hospital (Schwartz 2000). We found that when copollutants highly correlated with PM_2.5_ were included in the model, they tended to attenuate the magnitude and significance of its coefficient, except for mortality for those > 65 years of age. The latter finding suggests that, at least for deaths occurring in the elderly, gaseous copollutants do not confound the PM_2.5_–mortality associations. The gaseous pollutants, however, are spatially heterogeneous and may involve significant exposure misclassification. The separate effects of the gaseous pollutants on mortality will be the focus of subsequent analyses.

Overall, this large, multicounty analysis provides evidence of significant associations of PM_2.5_ with daily mortality among nearly two-thirds of California’s population.

## Figures and Tables

**Table 1:** Descriptive statistics for air pollutants and mortality in nine California counties, 1999–2002.

County	2000 population^a^	Days with data for PM_2.5_, temperature, and RH (*n*)	Mean daily PM_2.5_ (μg/m^3^)^b^	Mean daily temperature (°F)^b^	Mean daily RH (%)^b^	Mean daily all-cause mortality^b^
Contra Costa	949	698	14 (1–77)	60 (34–91)	64 (10–96)	16 (4–32)
Fresno	799	1,024	23 (1–160)	65 (35–94)	55 (18–96)	13 (3–28)
Kern	662	1,186	22 (1–155)	65 (36–95)	56 (13–100)	11 (2–25)
Los Angeles	9,519	1,221	21 (4–85)	64 (46–89)	57 (15–88)	146 (99–242)
Orange	2,846	682	21 (4–114)	63 (46–84)	67 (6–95)	40 (20–75)
Riverside	1,545	976	29 (2–120)	65 (43–90)	58 (6–100)	28 (9–63)
Sacramento	1,223	1,214	14 (1–108)	61 (36–91)	66 (13–100)	22 (7–45)
Santa Clara	1,683	717	15 (2–74)	59 (40–89)	69 (22–96)	22 (9–44)
San Diego	2,814	1,333	16 (0–66)	61 (43–84)	74 (16–100)	49 (26–87)

RH, relative humidity.

a In thousands.

b Values in parentheses indicate minimum–maximum.

**Table 2:** Mean daily deaths by mortality category in nine California counties, 1999–2002.

Mortality category	Contra Costa	Fresno	Kern	Los Angeles	Orange	Riverside	Sacramento	Santa Clara	San Diego
Age > 65 years	12.2	10.0	8.1	108.6	31.4	22.2	16.1	16.5	38.7
Male	7.2	6.4	5.4	70.7	18.3	13.8	10.4	10.3	23.7
Female	8.4	6.8	5.6	75.6	21.5	14.2	11.3	11.4	25.5
White non-Hispanic	12.3	9.3	8.6	86.0	32.7	23.1	16.8	15.5	39.6
Black non-Hispanic	1.5	0.8	0.6	20.6	0.4	1.3	2.0	0.5	2.1
Hispanic	0.9	2.4	1.5	26.7	3.6	3.0	1.3	2.5	4.9
In-hospital death	6.4	6.2	5.6	79.8	17.4	11.5	9.7	9.9	18.6
Out-of-hospital death	9.2	7.0	5.4	66.5	22.3	16.5	12.1	11.7	30.6
High school graduate	12.3	7.9	6.6	99.0	30.8	20.4	15.9	15.9	37.6
Not high school graduate	3.1	5.1	4.1	40.7	8.2	6.8	5.3	5.4	10.2
Diabetes	0.4	0.5	0.3	5.1	1.1	0.6	0.6	0.6	1.2
Cardiovascular disease	6.5	5.7	4.9	67.0	17.7	13.0	9.2	9.1	20.4
Ischemic heart disease	3.3	3.2	3.1	42.6	10.8	8.0	5.3	4.8	11.4
Respiratory disease	1.7	1.5	1.4	15.0	4.3	3.2	2.6	2.4	5.7

**Table 3:** Percent change in daily mortality categories and 95% CIs per 10-μg/m^3^ increment in PM_2.5_ using penalized splines and alternative lags [percent change (95% CI)].

County, lag^a^	All-cause mortality	Cardiovascular mortality	Respiratory mortality	Mortality > 65 years of age
Contra Costa				
2	0.8 (−1.0 to 2.6)	0.6 (−2.1 to 3.3)	0.4 (−5.1 to 6.0)	0.5 (−1.5 to 2.5)
01	0.4 (−1.9 to 2.7)	−0.6 (−4.1 to 2.9)	6.9 (0.1 to 13.8)	0.2 (−2.4 to 2.8)
Fresno				
2	0.3 (−0.8 to 1.4)	0.5 (−1.1 to 2.2)	1.2 (−1.8 to 4.2)	0.8 (−0.5 to 2.0)
01	0.2 (−1.1 to 1.5)	−0.1 (−2.1 to 1.9)	2.0 (−1.6 to 5.6)	0.4 (−1.1 to 1.9)
Kern				
2	−0.4 (−1.5 to 0.7)	0.8 (−0.6 to 2.3)	−1.2 (−3.9 to 1.5)	−0.1 (−1.4 to 1.1)
01	−0.3 (−1.5 to 0.9)	1.3 (−0.4 to 3.0)	−1.2 (−4.3 to 1.9)	−0.1 (−1.5 to 1.3)
Los Angeles				
2	−0.1 (−0.5 to 0.4)	0.1 (−0.6 to 0.8)	1.2 (−0.2 to 2.6)	−0.3 (−0.8 to 0.3)
01	0.6 (0.1 to 1.1)	0.4 (−0.3 to 1.2)	2.1 (0.6 to 3.6)	0.5 (−0.1 to 1.1)
Orange				
2	1.7 (0.6 to 2.9)	0.8 (−0.9 to 2.6)	5.7 (2.4 to 9.0)	1.2 (−0.1 to 2.5)
01	2.3 (1.0 to 3.6)	1.8 (−0.2 to 3.8)	7.6 (3.7 to 11.5)	2.3 (0.9 to 3.8)
Riverside				
2	−0.2 (−1.1 to 0.6)	0.0 (−1.2 to 1.2)	−0.5 (−2.7 to 1.7)	−0.3 (−1.3 to 0.6)
01	0.2 (−0.9 to 1.2)	−0.1 (−1.6 to 1.3)	−0.4 (−3.1 to 2.3)	0.1 (−1.0 to 1.3)
Sacramento				
2	0.8 (−0.4 to 2.0)	1.1 (−0.7 to 2.8)	3.5 (0.3 to 6.7)	0.9 (−0.4 to 2.3)
01	0.5 (−1.0 to 1.9)	0.9 (−1.2 to 3.0)	4.0 (−1.6 to 6.4)	1.1 (−0.6 to 2.8)
Santa Clara				
2	0.0 (−1.1 to 1.1)	−0.2 (−1.8 to 1.4)	1.7 (−1.6 to 5.0)	−0.2 (−1.4 to 1.0)
01	1.1 (−0.1 to 2.3)	1.1 (−0.6 to 2.9)	1.7 (−1.9 to 5.3)	1.2 (−0.1 to 2.6)
San Diego				
2	0.7 (−0.8 to 2.2)	1.0 (−1.3 to 3.2)	1.4 (−2.8 to 5.6)	1.4 (−0.3 to 3.0)
01	0.8 (−1.0 to 2.6)	0.3 (−2.2 to 2.9)	4.0 (−1.0 to 9.0)	1.2 (−0.8 to 3.2)
Pooled results				
2	0.2 (−0.2 to 0.7)	0.3 (−0.1 to 0.7)	1.3 (0.1 to 2.6)	0.2 (−0.2 to 0.7)
01	0.6 (0.2 to 1.0)	0.6 (0.0 to 1.1)	2.2 (0.6 to 3.9)	0.7 (0.2 to 1.1)

CI, confidence interval.

a Lag 01, average of 0- and 1-day lags of PM_2.5_; lag 2, 2-day lag of PM_2.5_. Model also includes day of week, spline smoothers of temperature and humidity, and two spline smoothers for time. Pooled results based on meta-analysis using a random-effects model.

**Table 4:** Pooled estimates of percent changes in daily mortality categories and 95% CIs per 10-μg/m^3^ increment in PM_2.5_ using natural splines.

Mortality category	df/year	% Change (95% CI)
All cause	4	0.5 (−0.1 to 1.1)
	8	0.4 (−0.1 to 0.9)
	12	0.3 (−0.1 to 0.7)
Cardiovascular	4	0.4 (−0.2 to 0.9)
	8	0.1 (−0.5 to 0.6)
	12	0.0 (−0.6 to 0.6)
Respiratory	4	2.1 (0.2 to 4.1)
	8	1.6 (−0.5 to 3.6)
	12	1.3 (−0.3 to 2.9)
Older than 65 years	4	0.7 (0.0 to 1.3)
	8	0.4 (−0.1 to 0.9)
	12	0.3 (−0.1 to 0.8)

Model includes average of 0- and 1-day lags of PM_2.5_, day of week, spline smoothers of temperature and humidity, and two spline smoothers of time. Pooled results based on meta-analysis using a random-effects model.

**Table 5:** Pooled estimates of percent changes in daily mortality categories and 95% CIs per 10-μg/m^3^ increment in PM_2.5_ using penalized splines.

Mortality category	% Change (95% CI)
All-cause	0.6 (0.2 to 1.0)
Cardiovascular	0.6 (0.0 to 1.1)
Respiratory	2.2 (0.6 to 3.9)
Age > 65 years	0.7 (0.2 to 1.1)
Ischemic heart disease	0.3 (−0.5 to 1.0)
Diabetes	2.4 (0.6 to 4.2)
Males	0.5 (−0.2 to 1.2)
Females	0.8 (0.3 to 1.3)
Whites	0.8 (0.2 to 1.3)
Blacks	0.1 (−0.9 to 1.2)
Hispanics	0.8 (−0.1 to 1.6)
In hospital	0.6 (−0.1 to 1.3)
Out of hospital	0.6 (0.1 to 1.1)
High school graduates	0.4 (0.0 to 0.8)
Non-high school graduates	0.9 (−0.1 to 1.9)

Model includes average of 0- and 1-day lags of PM_2.5_, day of week, spline smoothers of temperature and humidity, and two spline smoothers of time. Pooled results based on meta-analysis using a random-effects model.
